# Immunolocalization of leptin and leptin receptor in colorectal mucosa of ulcerative colitis, Crohn’s disease and control subjects with no inflammatory bowel disease

**DOI:** 10.1007/s00441-020-03297-4

**Published:** 2020-11-07

**Authors:** Flavia Merigo, Alessandro Brandolese, Sonia Facchin, Federico Boschi, Marzia Di Chio, Edoardo Savarino, Renata D’Incà, Giacomo Carlo Sturniolo, Andrea Sbarbati

**Affiliations:** 1 Department of Neuroscience, Biomedicine and Movement, Human Anatomy and Histology Section, University of Verona, 37134 Verona, Italy; 2 Department of Medicine, Gastroenterology Section, University of Verona, 37134 Verona, Italy; 3 Department of Surgery, Oncology and Gastroenterology, Gastroenterology Section, University Hospital of Padua, 35128 Padua, Italy; 4 Department of Computer Science, University of Verona, 37134 Verona, Italy; 5 Department of Diagnostic and Public Health, University of Verona, 37134 Verona, Italy

**Keywords:** Crohn’s disease, GLUT5, Leptin, Leptin receptor, Ulcerative colitis

## Abstract

The expression of leptin and leptin receptor (Ob-R) has been partially elucidated in colon of patients with inflammatory bowel diseases (IBDs), even though leptin is involved in angiogenesis and inflammation. We previously reported overexpression of GLUT5 fructose transporter, in aberrant clusters of lymphatic vessels in *lamina propria* of IBD and controls. Here, we examine leptin and Ob-R expression in the same biopsies. Specimens were obtained from patients with ulcerative colitis (UC), Crohn’s disease (CD) and controls who underwent screening for colorectal cancer, follow-up after polypectomy or with a history of lower gastrointestinal symptoms. Immunohistochemistry revealed leptin in apical and basolateral membranes of short epithelial portions, Ob-R on the apical pole of epithelial cells. Leptin and Ob-R were also identified in structures and cells scattered in the *lamina propria*. In UC, a significant correlation between leptin and Ob-R in the *lamina propria* was found in all inflamed samples, beyond non-inflamed samples of the proximal tract, while in CD, it was found in inflamed distal samples. Most of the leptin and Ob-R positive areas in the *lamina propria* were also GLUT5 immunoreactive in inflamed and non-inflamed mucosa. A significant correlation of leptin or Ob-R expression with GLUT5 was observed in the inflamed distal samples from UC. Our findings suggest that there are different sites of leptin and Ob-R expression in large intestine and those in *lamina propria* do not reflect the *status* of mucosal inflammation. The co-localization of leptin and/or Ob-R with GLUT5 may indicate concomitance effects in colorectal *lamina propria* areas.

## Introduction

Leptin is a 16 kDa hormone that is encoded by the *ob* gene. Initially believed to be mainly synthesized and secreted by adipocytes, leptin is now documented to be present in a variety of human and rodent tissues, including placenta, lung, muscle and kidney tissues, the olfactory system and gastric epithelia (Masuzaki et al. 1997; Wang et al. 1998).

Leptin acts through the leptin receptor (Ob-R) on the hypothalamus to regulate food intake and energy expenditure resulting in control of body weight and fat deposition. Mice lacking endogenous leptin (*ob/ob*) are genetically obese and develop hyperglycemia and hyperinsulinemia.

Ob-R is a member of the type I cytokine receptor family that is encoded by the *db* gene. Its deficiency causes obesity, hypogonadism, delayed onset of puberty, hypothyroidism and immune dysfunction (Mackey-Lawrence and Petri 2012).

Ob-R exists in five isoforms that differ in the length of the intracellular domain. Only the isoform that contains a full-length cytosolic domain (Ob-Rb) mediates the biological actions of leptin through the activation of multiple signaling pathways in parallel, including the JAK/STAT cascade, which is the major pathway used by leptin to exert its biological effects (Biorbaek et al. 1997; Attoub et al. 2000). Genetically deficient mice in the long form of Ob-R (*db*/*db* mice) are hyperphagic, obese and diabetic. The short isoforms are not yet characterized for their functional role although some of them are involved in the activation of signaling pathways (Francisco et al. 2018). Both the long and short isoforms have broad tissue distribution in rodents and humans, including in the small intestine and colon and cells of an adaptive and innate immune system (T cells, macrophages, natural killer cells, eosinophils, basophils and polymorphonuclear granulocytes).

In rodent studies, the expression of mRNA and protein for leptin was found in the gastric epithelium and glands of the fundic mucosa, whereas other sites of the gastrointestinal tract, including the small intestine, colon, rectum and pancreas lacked its expression (Bado et al. 1998).

In humans, leptin expression has been observed in gastric epithelial cells (Sobhani et al. 2000) and inflamed mucosa from colonic samples, localized at the apical membrane domain of epithelial cells. No leptin labeling has been found in normal colonic epithelial cells (Sitaraman et al. 2004).

Ob-R expression on intestinal cells has been mainly determined by western blot and quantitative PCR analysis in numerous previous studies. In rodents, Ob-R isoforms have been found mostly in the small intestine but also in stomach and colon. The functional leptin receptor Ob-Rb has been observed abundantly in the jejunum and to a lesser extent in the ileum (Morton et al. 1998), located on the apical and basal cytoplasm of jejunum enterocytes, and the colon (Barrenetxe et al. 2002). The multiple short isoforms have been found throughout the entire gastrointestinal tract but predominantly in the brush border membrane of jejunum epithelium (Buyse et al. 2001).

In human studies, OB-Rb has been described in the CACO-2 cell line and in normal duodenal enterocytes, located in the cytoplasm, basolateral plasma membrane and in the brush border membrane of epithelial cells (Barrenetxe et al. 2002). All Ob-R isoforms have been observed in colon cancer cell lines and colonic tissue, localized in the cytoplasm and cell membrane of epithelial cells (Hardwick et al. 2001).

The peripheral localization of leptin and Ob-R has stimulated the search for additional physiological roles, alongside their role in the regulation of energy balance, which is well documented. Nowadays, it is evident that leptin, like many other hormones, is multifunctional and contributes to the regulation of a variety of processes. As proven by vitro data and animal/human experiments, leptin is involved in glucose homeostasis and its secretion is stimulated by glucose uptake rather than circulating glucose and insulin levels (Welhoener et al. 2000). Leptin also inhibits insulin release from the pancreas in a dose-dependent manner acting as a signaling molecule between adipose tissue and the endocrine pancreas (Rossetti et al. 1997; Nemecz et al. 1999). It contributes to the inhibition of proinsulin synthesis, as well as in the reduction of secretions (Kieffer and Habener 2000; Lutz and Woods 2012; Park and Ahima 2015).

Leptin modulates immune and inflammatory responses. It is well known that it promotes macrophagic functions, expression of pro-inflammatory cytokines (interferon-γ or IFN-γ, tumor necrosis factor-α or TNF-α, IL-6, IL-1) and functional and morphological changes in human dendritic cells (Loffreda et al. 1998; Mattioli et al. 2005). On the contrary, its deficiency decreases production of TNF-α and sustains the production of the tumor growth factor-β (or TGF-β) by dendritic antigen-presenting cells, thus inducing T regulatory cell differentiation (Moraes-Vieira et al. 2014). In general, serum leptin levels are found to be increased during acute infection, inflammation and sepsis and inversely correlated with glucocorticoid levels (Zakrzewska et al. 1997).

Leptin and its receptor are also involved in acute and chronic diseases of intestine and especially inflammatory bowel disease (IBD) but the role of leptin in intestinal inflammation is still open for discussion as it is not yet clear whether leptin has anti- or pro-inflammatory properties or whether it is part of both pathways.

Rodent studies postulate that central or peripheral administration of leptin plays a gastroprotective action against various toxic agents and increases colonic cell proliferation (Attoub et al. 2000; Hardwick et al. 2001). In experimental colitis, exogenous leptin has been shown to have an anti-inflammatory function being able to inhibit tissue infiltration of neutrophils and thus protecting the colonic tissue from damage. This action partly depends on the release of glucocorticoids, which are considered the most potent endogenous inhibitors of inflammation (Cakir et al. 2004).

Conversely, leptin’s role in intestinal inflammation is generally assumed to be due to its modulatory effect on T cells (Siegmund et al. 2004), as demonstrated by the use of leptin antagonists that enhanced the presence of mucosal Treg cells and reduced the presence of mucosal inflammatory cytokines in an experimental model of colitis (Singh et al. 2013). In vivo, the addition of a β3 receptor agonist or a cholecystokinin-B receptor antagonist results in a reduction of leptin secretion and colonic damage during the early stages of colitis (Barbier et al. 2001). In rats, chemically induced acute intestinal inflammation is associated with elevated plasma leptin concentrations during the early stages of inflammation and is also correlated with the degree of inflammation (Barbier et al. 1998).

Available data on serum leptin concentrations in IBD patients are conflicting and do not help to unravel the intriguing question of whether the endogenous levels of leptin influence inflammatory processes. Tuzun et al. (2004) demonstrated that the serum leptin level is significantly higher in ulcerative colitis (UC) patients than in healthy subjects, with a more marked serum leptin level in UC patients with pancolitis, compared to those with a left-sided disease or procto-sigmoiditis. On the contrary, Karmiris et al. (2006) reported that the serum leptin level is decreased in both UC and Crohn’s disease (CD) patients when compared with healthy patients and is significantly associated with a body mass index (BMI) of < 25, as opposed to a BMI of ≥ 25. Nishi et al. (2005) stated that CD itself has no influence on the plasma concentration of leptin, which is not altered in CD patients and is not correlated with the degree of inflammation.

In IBD patients, prior investigations concerning the peripheral expression of leptin have been conducted in cell lines or limited to one segment of the colon, often used as a representative for the complete organ, neglecting, in this way, the different microenvironments of portions of the large intestine.

So far, to our knowledge, the expression and localization of leptin and Ob-R along the length of the large intestine have not yet been fully elucidated in native tissues. The literature data in this respect are heterogeneous and do not provide a conclusive picture of their expression, both in normal and IBD intestinal mucosa.

Our research group has recently identified the expression of the GLUT5 glucose transporter in colonic mucosal samples from UC and CD patients and controls (Merigo et al. 2018). We demonstrated that samples with histological evidence of both inflamed and non-inflamed tissues showed GLUT5 labeling in aberrant clusters of lymphatic vessels, suggesting a possible involvement of GLUT5 in lymphangiogenesis, which is a characteristic histological finding in the IBD pathogenesis.

Given the involvement of leptin in the proliferation and morphogenesis processes, we hypothesized that leptin and Ob-R might be expressed in the GLUT5 immunoreactive areas, thus showing to have a role in the inflammation and/or lymphangiogenesis process that characterizes the mucosa of IBD patients. Therefore, they could represent an attractive therapeutic target for human colon mucosa.

Accordingly, the aim of this study is to investigate the expression of leptin and Ob-R in the colorectal mucosa from IBD and non-IBD (control group) subjects, using the same samples in which the GLUT5 expression had previously been analyzed.

Firstly, we performed immunohistochemistry of leptin and Ob-R by light and confocal microscopy on biopsies of all traits of human large intestine.

Secondly, we compared the expression of leptin and Ob-R between them and with GLUT5 in adjacent sections and double-label experiments and then we quantified the samples with the combined immunoreactivities.

## Materials and methods

### Patients

Patients diagnosed with UC or CD and who were scheduled for diagnostic colonoscopy were enrolled. Patients who underwent colonoscopy for preventative screening of colorectal cancer or followed-up after polypectomy or with lower gastrointestinal symptoms were designated as the control group (CTRL).

The UC patient groups (*n* = 18) included 8 men and 10 women with a mean age of 47 ± SD 12.8 years (range 30–66) and a mean body mass index (BMI ± SD, weight in kg divided by height in m squared ± SD) of 24 ± 4.4 kg/m^2^ (range 18–32.9 kg/m^2^)_._

The CD patient group (*n* = 10) included 5 men and 5 women with a mean age of 37 ± 11.5 years (range 19–53) and a mean BMI 23.6 ± 2.5 kg/m^2^ (range 19.6–26.1 kg/m^2^).

The CTRL patient group (*n* = 16) included 6 men and 10 women with a mean age of 56 ± 13.2 years (range 27–72) and a mean BMI 24.8 ± 4.2 kg/m^2^ (range 18.1–31.1 kg/m^2^).

Inclusion criteria and patient characteristics are presented in Table 1.

**Table 1:** Clinicopathological data of patients

Patient information>		Patient group		
		UC	CD	CTRL
Age	Mean ± SD (years)	47 ± 12.8	37 ± 11.5	56 ± 13.2
Gender	Male	8	5	6
	Female	10	5	10
BMI	kg/m^2^, Mean ± SD	24 ± 4.4	23.6 ± 2.5	24.8 ± 4.2
	BMI ≥ 30 (*n*)	2	0	2
	BMI 25–29 (*n*)	4	3	7
	BMI < 25 (*n*)	12	7	7
**Patients enrolment criteria (to colonoscopy)**				
Periodic follow-up (*n*)		16	2	3^*^
New stadiation of BD (*n*)		2	8	-
Abdominal pain (*n*)		-	-	5
Chronic Diarrhea (*n*)		-	-	4
Colorectal Cancer Screening (*n*)		-	-	2
Stipsis (*n*)		-	-	1
Hemorrhoids (*n*)		-	-	1

*Follow up after polypectomy. UC: Ulcerative Colitis; CD: Crohn’s Disease; CTRL: Controls

### Colorectal samples

Colorectal biopsies were obtained from patients undergoing endoscopic colonoscopy or recto-sigmoidoscopy (2 in the CTRL group). All samples of the large intestine tracts were taken for diagnostic purposes, according to the endoscopist’s judgment and for immunohistochemistry (IHC). The samples were collected from adjacent sites to compare the level of inflammation in independent samples. In 14 (8 in UC, 1 in CD, 5 in CTRL) of the 44 patients who underwent a complete colonoscopy, biopsies were obtained from all 6 portions of the colon-rectum (cecum, ascending colon, transverse, descending, sigmoid colon, rectum). In the remaining 30 patients, biopsies were only obtained from certain endoscopically examined portions.

A total of 147 biopsies of colorectal mucosa were collected for IHC analysis. Inflammatory mucosal *status* at the sampling site was evaluated endoscopically in all biopsies and histologically in 127 out of 147 biopsies by an experienced pathologist, who evaluated the mononuclear and polymorphonuclear cell infiltration of the mucosal layer. In the event of endoscopic findings, inflammatory *status* was graded according to the Mayo endoscopic score in the UC patients (Mayo score of 0 indicates normal colonic mucosa and > 0 indicates evidence of macroscopic active inflammation). However, with respect to previously resected CD (3 out of 10) patients, inflammatory *status* was graded according to the Rutgeerts score (Rutgeerts score of 0–1 indicates normal ileocolic anastomosis and > 1 macroscopic relapse of CD). For the non-resected CD and CTRL patients, inflammatory mucosal *status* was graded as documented in the endoscopic report. Based on histological and endoscopic grading, their inflammatory *status* was determined and classified as inflamed or non-inflamed. When the endoscopic grade differed from the histological grade, the final biopsy *status* was decided on the basis of the pathologist’s assessment. 

The complete list of all biopsies analyzed by IHC, classified according to inflammation *status*, is shown in Table 2.

**Table 2:** Biopsies analyzed by immunohistochemistry in all large intestine tracts

Large intestine		Status of patient biopsies					
		UD		CD		CTRL	
		Inflamed (*n*)	Non-inflamed (*n*)	Inflamed (*n*)	Non-inflamed (*n*)	Inflamed (*n*)	Non-inflamed (*n*)
Proximal Colon	Cecum	2	7	0	1	2	7
	Ascending	6	9	4	1	4	5
	Transverse	4	5	3	0	3	6
Distal Colon	Descending	6	6	4	1	2	6
	Sigmoid	7	3	4	2	4	7
	Rectum	7	3	4	1	4	7

UC: Ulcerative Colitis; CD: Crohn’s Disease; CTRL: Controls

### Tissue preparation

After sampling, specimens were fixed in 40 g/L of formaldehyde and processed by embedding them in paraffin using standard methods. Sections were cut to 7 µm of thickness, mounted on polylysine-coated microscope slides and processed for immunoperoxidase and double immunofluorescence labeling. The immunostaining of proteins included in this study was observed in adjacent sections of each sample. The evaluation of immunoreactivity was performed by two investigators without knowledge of the samples origin. Primary and secondary antibodies used for IHC are listed in Table 3.

**Table 3:** Primary and secondary antibodies used for immunohistochemistry

**Antibody**	**Host**	**Dilution**	**Source**
Anti-Leptin	Rabbit	1:400	cat#ab3583, abcam, Cambridge, UK
Anti-leptin receptor (Ob-R)	Rabbit	1:200	cat#ab5593, abcam
Anti-GLUTS	Rabbit	1:400	cat#ab36057, abcam
Anti-LYVE-1	Rabbit	1:500	cat#Bs-1311R, Bioss, Woburn, MA, USA
Anti-VEGF	Mouse	1:50	cat#GTX83426, GeneTex, Irvine, CA, USA
Polyclonal anti-rabbit IgG/biotinylated	Swine	1:400	cat#E0353, Dako, Milan, Italy
Cy™3-Fab fragment anti-Rabbit IgG	Goat	1:100	cat#111-167, Jackson Lab., Baltimore, MD, USA
FITC-Fab fragment anti-rabbit IgG	Goat	1:200	cat#111-097, Jackson Lab.
FITC-Fab fragment anti-mouse IgG	Goat	1:200	cat#315-097-003, Jackson Lab.
Unconjugated Fab fragment anti-rabbit IgG	Goat	1:30	cat#111-007, Jackson Lab.

### Peroxidase-Immunohistochemistry (IHC)

Sections were deparaffinized in xylene and then rehydrated with passages in alcohol in decreasing concentrations to water. Dewaxed sections were microwaved for 15 min in 10 mM of citrate buffer (pH 6.0) for antigen retrieval. Endogenous peroxidase activity was blocked by incubating the slides in 30 mL/L H_2_O_2_ in methanol for 30 min. After rinsing in PBS, the slides were first incubated with blocking solution (3 mL/L Triton X-100, 10 g/L bovine serum albumin, and 10 mL/L normal swine or rabbit serum); the solution was used to dilute the antibodies. Subsequently, the sections were incubated with primary antibodies overnight at 4 °C and then reacted with biotinylated secondary antibody for 1 h at room temperature. The immunoreaction was detected using a Vectastain Elite ABC kit (Vector Laboratories, Burlingame, CA, USA) and then visualized with 3.3′-diaminobenzidine tetrahydrochloride (Dako, Milan, Italy) for 5–10 min. Finally, the sections were mounted in Entellan (Merck, Milan, Italy).

Negative controls were sections that were processed as above but with the omission of the primary antibody. Control sections for Ob-R were also prepared by preabsorbing the primary antibodies with the corresponding peptide (5 μg/1 mL of antibody; LeptinR peptide, cat#ab5837, abcam). Sections of rat adipose tissue were used as positive controls for leptin and Ob-R antibodies.

### Light microscopy

Sections were examined with an Olympus BX51 microscope (Olympus, Tokyo, Japan) equipped with a digital camera (DKY-F58 CCD JVC, Yokohama, Japan). Digital images were analyzed and processed with Image-ProPlus 7.0 software (Media Cybernetics, Silver Spring, MD, USA). Images were composed with Adobe Photoshop software (v. 6.0; Adobe Systems, Mountain View, CA, USA) to regulate contrast and brightness.

### Immunofluorescence microscopy

Double immunofluorescence staining was used to compare immunolabeling of leptin and Ob-R with GLUT5, with the lymphatic vessel endothelial hyaluronan receptor 1 (LYVE-1), a marker for lymphatic vessel endothelium in humans and rodents and with the vascular endothelial growth factor (VEGF), a marker for vascular endothelial cells.

The double-label assay was carried out sequentially using a method that relied on the use of secondary monovalent Fab fragments because all primary antibodies are raised in the same species (Lewis Carl et al. 1993; Negoescu et al. 1994). Paraffin sections (processed as described above) were used for double staining, which was performed as described by Merigo et al. (2011). After completing the staining protocol, the sections were immersed with 1 g/L Sudan Black B (Merck) in 70% ethanol for 20 min at room temperature to reduce tissue autofluorescence. After rinsing, the sections were counterstained with TO-PRO 3 (Life Technologies, Monza, Italia), mounted in glycerol/PBS and observed with a Zeiss LSM 710 confocal microscope equipped with argon (488 nm), helium/neon (543 nm) and helium/neon (633 nm) excitation beams (Zeiss, Jena, Germany). Sequential acquisition, i.e., one color at a time, was utilized on double-labeled tissues to avoid side-band excitation of the inappropriate fluorophore. All images for publication were compiled using Adobe Photoshop software, adjusting only brightness and contrast.

Control sections were prepared using one of the following methods: (1) replacing the second primary antibody with normal rabbit serum (Dako, Milan, Italy), (2) exchanging the fluorophores of the secondary antibodies, (3) omitting the primary antibody, or (4) changing the sequence of the secondary antibody application.

### Statistical analysis

In order to evaluate the differences between the expression of each protein in the collected samples, the Fisher’s exact test was applied. Specifically, we compared the expression of leptin vs Ob-R, leptin vs GLUT5 and Ob-R vs GLUT5. Differences were considered statistically significant at the 5% significance level (*P* < 0.05).

## Results

As with our previous study (Merigo et al. 2018), the IHC results of samples from the cecum, ascending and transverse colon were grouped together as data of the proximal tract; those of samples from the descending, sigmoid colon and rectum were grouped as data of the distal tract of the large intestine.

### Immunolocalization of leptin in colorectal samples in IBD and control group

**Fig. 1:**
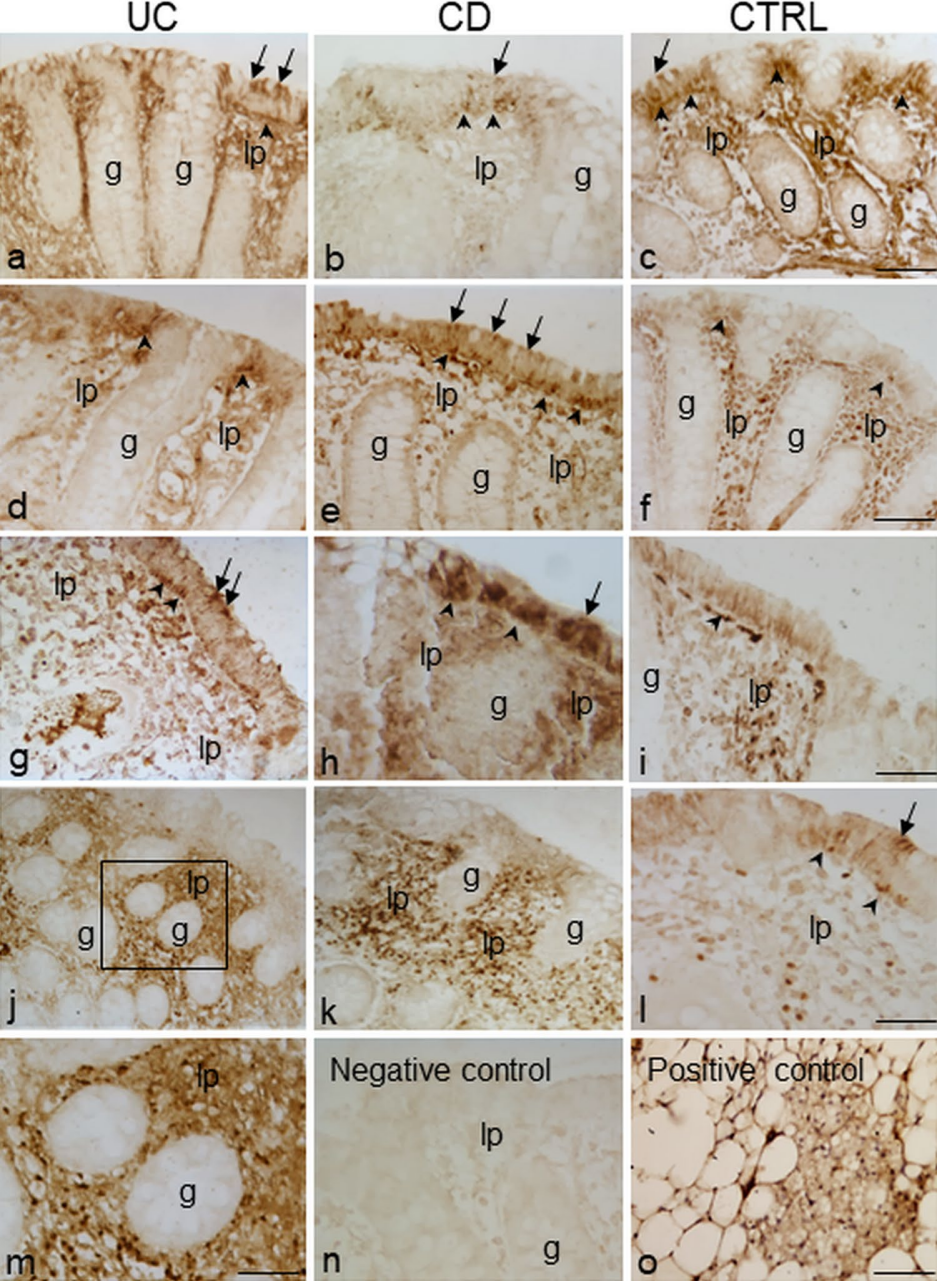
Immunoperoxidase staining showing leptin immunoreactivity in samples of the proximal (**a**–**c**; **g**–**i**) and distal (**d**–**f**; **j**–**l**) tract of the human large intestine from ulcerative colitis (UC), Crohn’s disease (CD) and control (CTRL) patients. The mucosa is non-inflamed in **a**–**f** and inflamed in **g**–**l**. Apical (arrows) and basal (arrowheads) epithelial immunostaining is visible. The boxed area in **j** is shown at higher magnification in **m**. No staining is observed when leptin antibody was omitted (**n**). Specific staining is observed in a section of rat adipose tissue used as positive control. lp *lamina propria*, g gland. Bars 30 µm (**c**,** f**,** i**, **l**, **o**), 15 µm (**m**)

Distinct expression patterns of leptin staining were observed throughout portions of the large intestine. Leptin expression was found both on the epithelium and *lamina propria*. Epithelial immunostaining was heterogeneous and concentrated on the apical or basal cytoplasm (Fig. 1a-–i, arrows and arrowheads, respectively) and/or extended throughout the cell. In only some cases, leptin labeling was also detected on the brush border membrane. Nevertheless, these positivities were observed only in restricted areas of the epithelium. Similar findings were observed throughout the entire tract of the large intestine, in both non-inflamed (Fig. 1a-–f) and inflamed (Fig. 1g-–l) mucosal tissue from each patient group. Samples with leptin-negative epithelium were also observed in each group of patients.

Frequently, epithelial positivity was associated with positivity in the underlying *lamina propria*, in which leptin-positive areas were well distinguishable (Fig. 1a-l). Inside the leptin-positive areas, scattered cells and vessels were the structures predominantly labeled (Fig. 1m).

The labeling intensity, the extension and the number of the leptin-immunoreactive areas varied among samples. In some cases, leptin staining was distributed throughout the entire *lamina propria*; in others, it was absent.

No specific labeling was seen in the control sections when immunohistochemistry was performed without the primary antibody (Fig. 1n). Positive control section of rat adipose tissue displayed intense leptin staining (Fig. 1o).

Leptin in *lamina propria* was detected in both IBD and control samples; the number of samples with positive staining was counted and their percentage was calculated (Table 4). In UC patients, the percentage of leptin-positive samples was similar in inflamed and non-inflamed samples from the proximal (75% vs 76.2%) and the distal tract (75% vs 83.3%), respectively. In the CD group, the percentage of positive samples was higher in the non-inflamed samples than in the inflamed samples from both the proximal (100% vs 71.4%) and the distal tract (100% vs 75%), respectively. In the CTRL group, the percentage of positive samples was higher in the non-inflamed samples than in the inflamed samples from the proximal tract (94.4% vs 77.8%) and higher in the inflamed samples than the non-inflamed samples from the distal tract (100% vs 85%).

**Table 4:** Percentage of samples with leptin and Ob-R expression in *lamina propria *of large intestine biopsies

**Patient group**	**LARGE INTESTINE**					
	**Biopsy Status**	**Proximal Tract**		**Biopsy status**	**Distal Tract**	
		**Leptin** (%)	**Ob-R** (%)		**Leptin** (%)	**Ob-R** (%)
UC	Inflamed (*n* = 12)	75.0	66.7	Inflamed (*n* = 20)	75.0	55.0
	Non-inflamed (*n* = 21)	76.2	42.9	Non-inflamed (*n* = 12)	83.3	91.7
CD	Inflamed (*n* = 7)	71.4	42.9	Inflamed (*n* = 12)	75.0	66.7
	Non-inflamed (*n* = 2)	100	50.0	Non-inflamed (*n* = 4)	100	100
CTRL	Inflamed (*n* = 9)	77.8	66.7	Inflamed (*n* = 10)	100	70.0
	Non-Inflamed (*n* = 18)	94.4	83.3	Non-Inflamed (*n* = 20)	85.0	80.0

UC: Ulcerative colitis; CD: Crohn’s disease; CTRL: controls

### Immunolocalization of Ob-R in colorectal samples in IBD and control group

**Fig. 2:**
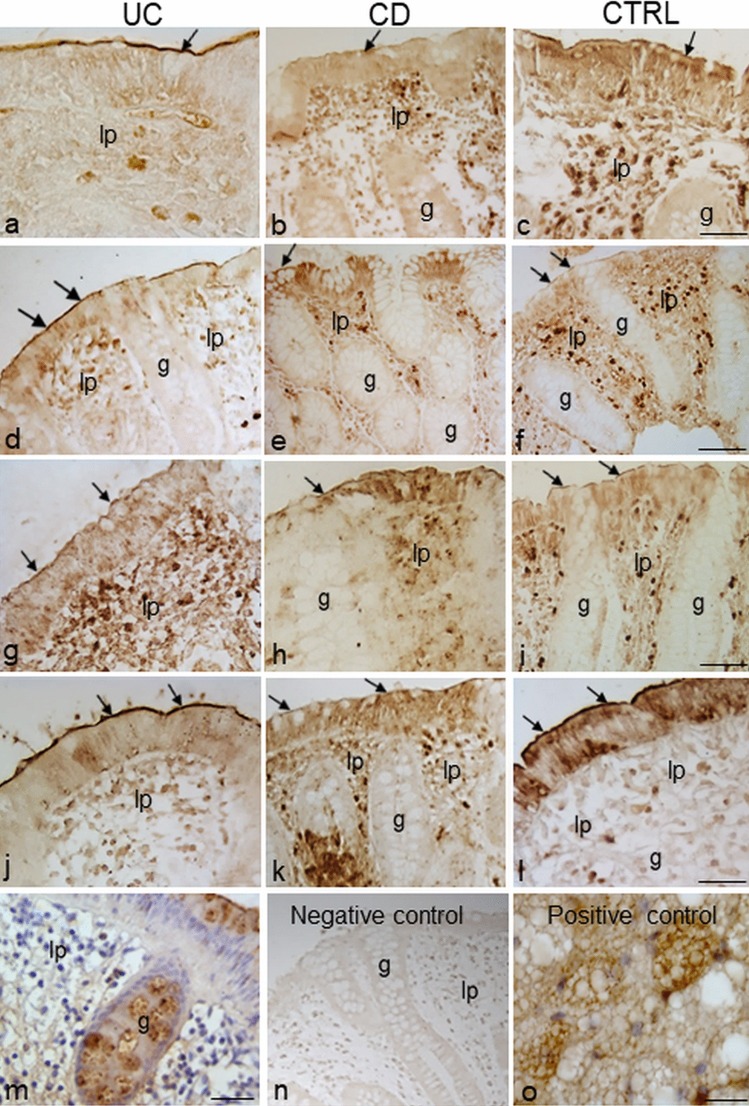
Immunoperoxidase staining showing Ob-R immunoreactivity in samples of the proximal (**a**–**c**; **g**–**i**) and distal (**d**–**f**; **j**–**m**) tract of the human large intestine from ulcerative colitis (UC), Crohn’s disease (CD) and control (CTRL) patients. The mucosa is non-inflamed in **a**–**f**, **m** and inflamed in **g**–**l**. Brush border (arrows) epithelial immunostaining is visible. No staining is observed when Ob-R antibody was omitted (**n**). Specific staining is observed in section of rat adipose tissue used as positive control. lp *lamina propria*, g gland. Bars 30 µm (**c**, **f**, **i**, **l**), 10 µm (**m**, **o**)

Immunoperoxidase and immunofluorescent labeling revealed Ob-R expression in the epithelium, localized on the brush border at the apical cell membrane in all samples (Fig. 2a-l, arrows). It was also found in the basolateral plasma membrane and cytoplasm of epithelial cells (Fig. 2l). Occasionally, Ob-R expression was observed with a granular appearance within the cytoplasm of the goblet cells both in the epithelium and crypts of the *lamina propria* (Fig. 2m). No differences between the proximal and the distal tract were noted.

In *lamina propria*, Ob-R immunoreactivity was present in structures and cells scattered in the connective tissue. The immunoreactive cells appeared intensely marked, round in shape and were generally located close to vessels that appeared in a non-round shape and with a dilated lumen (Fig. 2a-l). As for their morphological characteristics, the vessels were identified as lymphatic vessels. The intensity and distribution of the staining pattern were variable among the different samples. No Ob-R labeling was detected in some samples.

No specific labeling was seen in the control sections when immunohistochemistry was performed without the primary antibody or when Ob-R antibody was preincubated with the corresponding antigen peptide (Fig. 2n). A positive control section of rat adipose tissue displayed intense Ob-R staining (Fig. 2o).

Ob-R immunoreactivity in the *lamina propria* was observed throughout samples of the entire tract of the large intestine, both in non-inflamed (Fig. 2a-f) and inflamed (Fig. 2g-l) mucosal tissue from each patient group (Table 4). In the UC group, the percentage of Ob-R-positive samples was higher in the inflamed samples than in the non-inflamed samples from the proximal tract (66.7% vs 42.9%), whereas the percentage was higher in the non-inflamed samples as compared to the inflamed samples from the distal tract (91.7% vs 55%). In the CD and CTRL patients, the percentage of Ob-R immunoreactive samples was higher in the non-inflamed samples as compared to the inflamed samples both from the proximal tract (50% vs 42.9% for CD; 83.3% vs 66.7% for CTRL) and the distal tract (100% vs 66.7% for CD; 80% vs 70% for CTRL).

### Comparison and colocalization of leptin and Ob-R expression in *lamina propria*

**Fig. 3:**
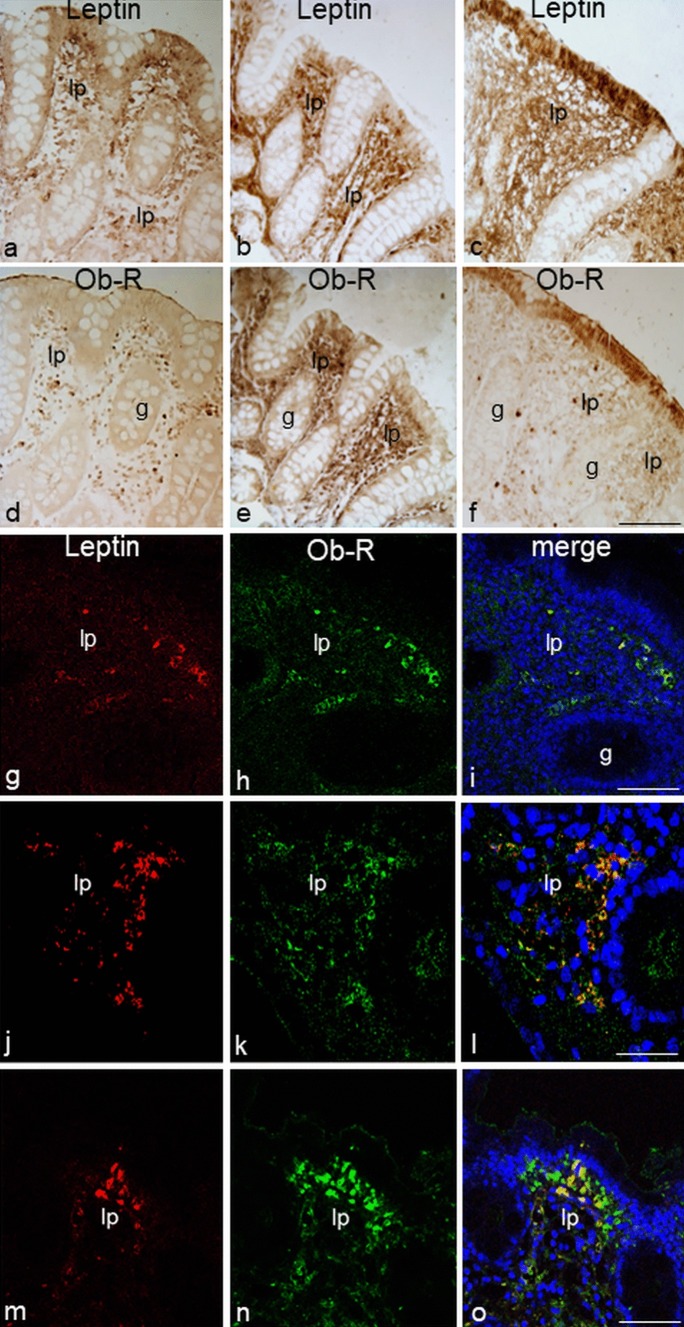
Immunoperoxidase staining (**a**–**f**) showing leptin and Ob-R immunoreactivity in adjacent sections of the distal tract of large intestine from ulcerative colitis (**a**, **d**), Crohn’s disease (**b**, **e**) and control (**c**, **f**) patients. In all samples, the mucosa is non-inflamed. Double-immunofluorescent confocal microscopy (**g**–**o**) showing expression of leptin (red) with Ob-R (green) in samples of the distal tract of large intestine from ulcerative colitis (**g**–**i**), Crohn’s disease (**j**–**l**) and control (**m**–**o**) patients. In all samples, the mucosa is inflamed. Counterstained with TO-PRO-3 (blue). lp *lamina propria*, g gland. Bars 30 µm (**f**), 50 µm (**i**, **o**), 20 µm (**l**)

Using immunoperoxidase labeling on adjacent sections of each sample and the double-label immunofluorescence technique, we compared the expression of leptin and Ob-R in *lamina propria* to quantify the number of samples with both immunoreactivities. We considered samples to be leptin and Ob-R positive (Lept^+^/Ob-R^+^) if they were consistent with both immunoreactivities localized in corresponding areas of contiguous sections. Ob-R labeling was detected in the leptin-positive areas in most samples (Lept^+^/Ob-R^+^) but with some differences in the expression pattern. The staining level of leptin was generally more marked than with Ob-R in adjacent sections (Fig. 3a-f) but opposite staining was found in some sections (Fig. 3g-o). We also observed Ob-R labeling in areas that did not show leptin expression (Lept^−^/Ob-R^+^ sample), or areas with opposite staining (Lept^+^/Ob-R^−^ sample). Absence of both expressions in adjacent sections of some samples was found (Lept^−^/Ob-R^−^).

No specific double-labeling was seen when the second antibody was replaced with normal rabbit serum.

The percentage of samples with associated leptin/Ob-R expression is shown in Table 5**.** The percentage of (Lept^+^/Ob-R^+^) samples was always higher with respect to the others (i.e., Lept^+^/Ob-R^−^, Lept^−^/Ob-R^+^), in inflamed and non-inflamed samples from the proximal and distal tract of all patient groups, with the exception of CD patients, in which all non-inflamed samples from the proximal tract were Lept^+^/Ob-R^−^. Hence, the expression of leptin and Ob-R was significantly correlated (*P* < 0.05) in all inflamed samples of proximal and distal tracts from UC patients and was similar in non-inflamed samples of the proximal tract from UC patients (Table 5). Likewise, a significant correlation (*P* < 0.05) between leptin and Ob-R expression was demonstrated in inflamed samples of the distal tract from CD patients (Table 5).

**Table 5:** Percentage of samples with leptin (Lept)/Ob-R expression in *lamina propria* structures of large intestine biopsies

**Patient group**		**LARGE INTESTINE**						
		PROXIMAL TRACT				DISTAL TRACT		
	**Biopsy status**	**Lept**^+^**/Ob-R**^+^ (%)	**Lept**^+^**/Ob-R**^−^ (%)	**Lept**^−^**/Ob-R**^−^ (%)	**Biopsy status**	**Lept**^+^**/Ob-R**^+^ (%)	**Lept**^+^**/Ob-R**^−^ (%)	**Lept**^-^**/Ob-R**^−^ (%)
UC	Inflamed (*n* = 12)	**66.7**	8.3	25.0	Inflamed (n = 20)	**55.0**	20.0	25.0
	Non-inflamed (n = 21)	**42.8**	33.3	23.9	Non-inflamed (n = 12)	74. 9	8.3	0.0 *
CD	Inflamed (n = 7)	42.8	28.6	28.6	Inflamed (n = 12)	**66.7**	8.3	25.0
	Non-inflamed (*n* = 2)	0.0	100	0.0	Non-inflamed (*n* = 4)	100	0.0	0.0
CTRL	Inflamed (*n* = 9)	55.5	22. 2	11.1*	Inflamed (*n* = 10)	70.0	30.0	0.0
	Non-inflamed (*n* = 18)	83.3	11.1	5.6	Non-inflamed (*n* = 20)	75.0	10.0	10.0 *

UC: ulcerative colitis; CD: Crohn’s disease; CTRL: controls *indicates the group in which the residual percentage of samples is Lept^−^/Ob-R^ +^ Percentages in bold indicate significant correlation between the expression of Lept and Ob-R

### Leptin and Ob-R immunoreactive samples in biopsy groups subdivided by patient BMI

The expression of leptin and Ob-R in *lamina propria* was also evaluated in sample groups obtained by classifying the patients according to their BMI.

For all patient groups, the percentage of leptin (Table 6) and Ob-R (Table 7) immunolabelled samples was higher in patients with a normal weight than in overweight and obese patients. There was no difference between inflamed or non-inflamed samples and the proximal and distal tract. The only relevant difference regarding the control group was that inflamed leptin-positive samples from the distal tract were found in 60% of overweight patients and 40% of patients with a normal weight.

**Table 6:** Percentage of immunoreactive samples for leptin expression in *lamina propria *structures of biopsy groups subdivided by patient body mass index

Patient group	LARGE INTESTINE							
	Proximal tract				Distal tract			
		BMI of patients				BMI of patients		
	Biopsy status	Normal weight (%)	Overweight (%)	Obese (%)	Biopsy status	Normal weight (%)	Overweight (%)	Obese (%)
UC	Inflamed	58.3	16.7	0.0	Inflamed	45.0	25.0	5.0
	Non-inflamed	47.7	19.0	9.5	Non-inflamed	50.0	25.0	8.3
CD	Inflamed	42.9	28.5	0.0	Inflamed	50.0	25.0	0.0
	Non-inflamed	50.0	50.0	0.0	Non-inflamed	50.0	50.0	0.0
CTRL	Inflamed	55.6	22.2	0.0	Inflamed	40.0	60.0	0.0
	Non-inflamed	44.4	33.3	16.7	Non-inflamed	50.0	20.0	15.0

UC: Ulcerative Colitis; CD: Crohn’s Disease; CTRL: Controls

**Table 7:** Percentage of immunoreactive samples for Ob-R expression in *`*structures of biopsy groups subdivided by patient body mass index

Patient group	LARGE INTESTINE							
	PROXIMAL TRACT				DISTAL TRACT			
		BMI of patients				BMI of patients		
	Biopsy status	Normal weight (%)	Overweight (%)	Obese (%)	Biopsy status	Normal weight (%)	Overweight (%)	Obese (%)
UC	Inflamed	58.3	8.4	0.0	Inflamed	40.0	10.0	5.0
	Non-inflamed	33.3	4.8	4.8	Non-inflamed	58. 3	25.0	8.4
CD	Inflamed	14.4	28.6	0.0	Inflamed	50.0	16.7	0.0
	Non-inflamed	50.0	0.0	0.0	Non-inflamed	50 .0	50.0	0.0
CTRL	Inflamed	44.5	22.2	0.0	Inflamed	60.0	10.0	0.0
	Non-inflamed	38.9	27.7	16.7	Non-inflamed	40.0	20.0	20.0

UC: Ulcerative Colitis; CD: Crohn’s Disease; CTRL: Controls

### Comparison of leptin and/or Ob-R expression with GLUT5, Lyve-1 and VEGF immunoreactivity in* lamina propria*

To further characterize the leptin/Ob-R expression in the *lamina propria*, we compared the distribution of leptin and Ob-R with GLUT5, Lyve-1 and VEGF.

#### Colocalization of leptin and GLUT5

**Fig. 4:**
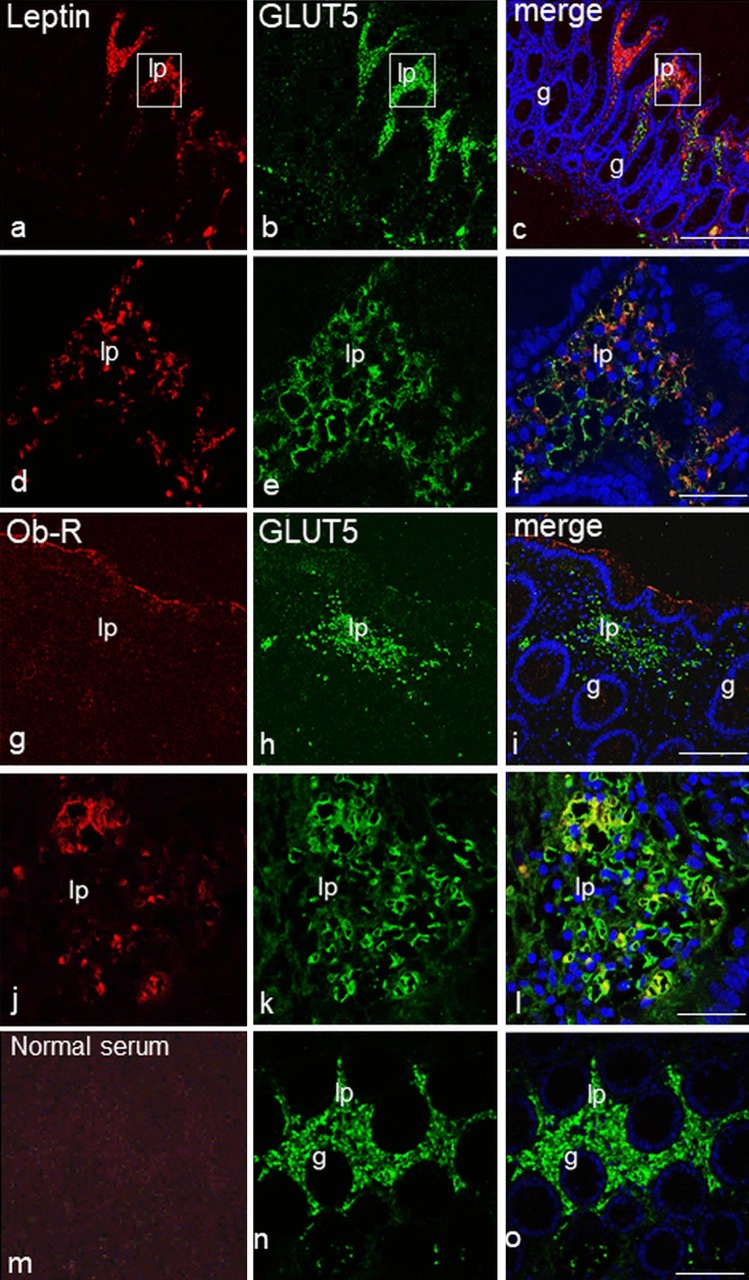
Double-immunofluorescent confocal microscopy showing expression of leptin or Ob-R (red) with GLUT5 (green) in a sample of the proximal tract of large intestine with non-inflamed mucosa from a control subject (**a**–**f**) and in samples of the distal tract with inflamed mucosa from ulcerative colitis patients (**g**–**l**). The boxed area in **a**–**c** is shown at higher magnification in **d**–**f**, respectively. No specific staining is observed when Ob-R antibody was replaced with normal serum (**m**–**o**). Counterstained with TO-PRO-3 (blue). lp *lamina propria*, g gland. Bars 100 µm (**c**, **i**), 20 µm (**f**,** l**), 50 µm (**o**)

As expected, light immunohistochemistry and double immunofluorescence microscopic revealed the presence of GLUT5 in large areas of the *lamina propria*; these findings are in agreement with those of the previous work (Merigo et al. 2018). We considered leptin- and GLUT5-positive samples (Lept^+^/GLUT5^+^) when both the immunoreactivities were present in contiguous sections and when labeling was localized in corresponding areas. In this case, we frequently found that staining was not always coincident, as some leptin-positive areas were more extensive than those with GLUT5 expression, whereas with others we found the opposite (Fig. 4a-f). No correlation between the two staining intensities was observed because an intense GLUT5 labeling could be associated indifferently with an intense or weak leptin staining. Leptin-positive samples that lacked GLUT5 expression (Lept^+^/GLUT5^−^) and GLUT5-positive samples that lacked leptin expression (Lept^−^/GLUT5^+^) were also observed. In some samples, no leptin or GLUT5 labeling was seen (Lept^−^/GLUT5^−^).

GLUT5 and leptin staining was observed in both inflamed and non-inflamed tissue samples.

**Fig. 5:**
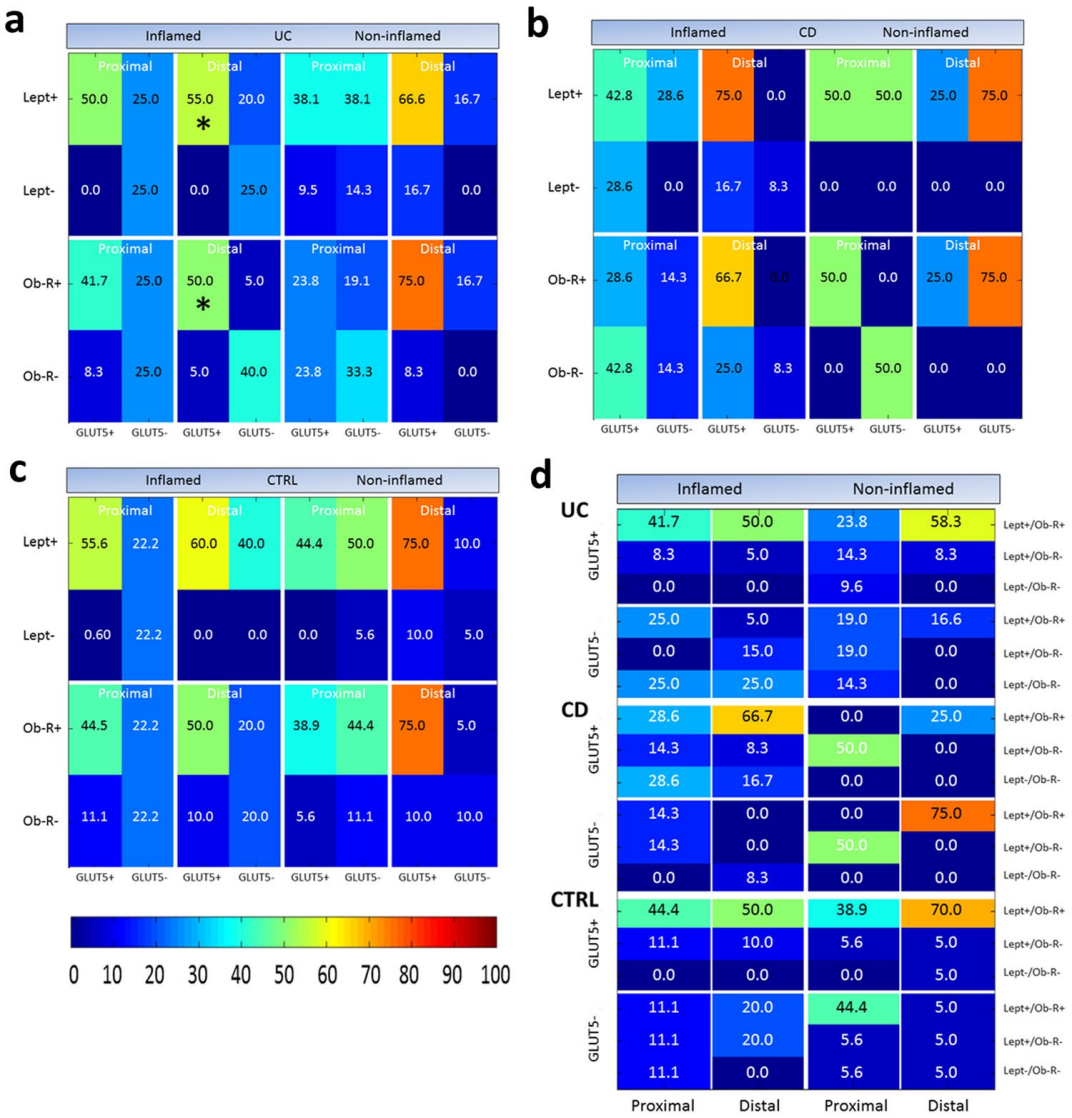
Chromatic representations of the percentages of leptin (Lept), Ob-R and GLUT5 positive and negative samples in *lamina propria* tissue samples from the proximal and distal tract of large intestine of patients with ulcerative colitis (UC, **a**), Crohn’s disease (CD, **b**) and controls (CTRL, **c**). **a**–**c** Numbers represent the percentages of both inflamed and non-inflamed mucosa samples from each study group with Lept or Ob-R expression associated with the expression of GLUT5; **d** Numbers show combined expression (Lept/Ob-R) associated to those of GLUT5 in both inflamed and non-inflamed mucosa samples from each study group. The percentage is visually highlighted with a color; the color bar (below **c**) indicates the correspondence between color and percentage value. Asterisks indicate a significant correlation between the expressions represented

The percentage of samples with both immunoreactivities is shown in Fig. 5(a) for the UC, Fig. 5(b) for the CD and Fig. 5(c) for the CTRL group.

#### Colocalization of Ob-R and GLUT5

We also compared Ob-R and GLUT5 expression and, as reported previously, we considered Ob-R and GLUT5 positive samples (Ob-R^+^/GLUT5^+^) when both the immunoreactivities were present in contiguous sections and labeling was localized in corresponding areas of the *lamina propria*. No correlation between the intensity of immunolabeling was observed because an intense GLUT5 labeling could be associated with a negative or intense or weak Ob-R staining (Fig. 4g-l). In control sections, no specific double-labeling was seen when the second primary antibody was replaced with normal rabbit serum (Fig. 4m-o). However, Ob-R immunoreactivity did not necessarily imply the presence of GLUT5 immunoreactive clusters because positive Ob-R areas could correspond to GLUT5 negative areas (Ob-R^+^/GLUT5^−^) and vice versa (Ob-R^−^/GLUT5^+^). A reduced number of samples lacked both Ob-R and GLUT5 expression (Ob-R^−^/GLUT5^−^).

The percentage of samples with both immunoreactivities is shown in Fig. 5(a) for the UC group, Fig. 5(b) for the CD group and Fig. 5(c) for the CTRL group.

The expression of leptin or Ob-R showed a significant correlation with that of GLUT5 (*P* < 0.05) in the inflamed samples of the distal tract from UC patients (Fig. 5a, asterisk). In other patient groups, there was no significant correlation found.

#### Colocalization of leptin and Ob-R with GLUT5

**Fig. 6:**
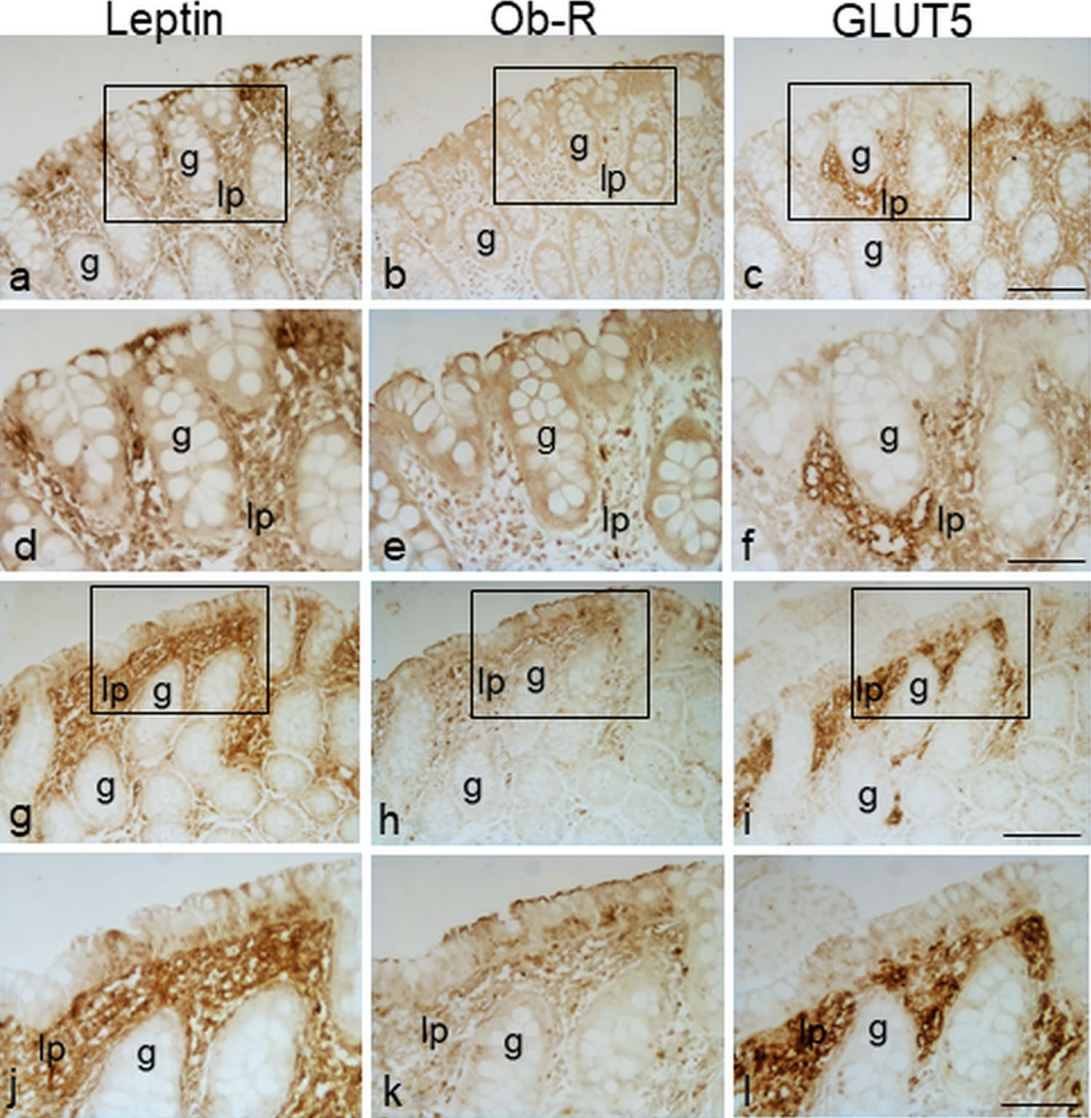
Immunoperoxidase staining showing leptin, Ob-R and GLUT5 immunoreactivity in adjacent sections of distal large intestine from ulcerative colitis (**a**–**f**) and control (**g**–**l**) subjects. In all samples, the mucosa is non-inflamed. The boxed area in **a**–**c** and **g**–**i** is shown at higher magnification in **d**–**f** and **j**–**l**, respectively. lp *lamina propria*, g gland. Bars 60 µm (**c**, **i**), 30 µm (**f**, **l**)

To better investigate the localization of the three antibodies in individual samples, we compared their immunoreactivity by immunoperoxidase labeling in contiguous sections. Light immunohistochemistry revealed the expression of GLUT5 immunoreactivity in sections that were also leptin and Ob-R positive (Lept^+^/Ob-R^+^/GLUT5^+^), which is consistent with previous results (Fig. 6a-l).

Furthermore, in some samples, we observed GLUT5 labeling in sections that were leptin positive and Ob-R negative (Lept^+^/Ob-R^−^/GLUT5^+^), or leptin negative but Ob-R positive (Lept^−^/Ob-R^+^/GLUT5^+^).

As shown in Fig. 5(d), the Lept^+^/Ob-R^+^/GLUT5^+^ samples were those in greater percentages than other possible combinations both in the proximal and distal tract of the UC and CTRL group, with the most evident differences being between the proximal and distal tract of the non-inflamed samples. In the CD group, the highest percentage of samples from the distal tract exhibited immunoreactivity for leptin and Ob-R but without the expression of GLUT5 (Lept^+^/Ob-R^+^/GLUT5^−^).

#### Colocalization of leptin and Ob-R with Lyve-1 or VEGF

Leptin or Ob-R expression was compared with expression of Lyve-1 and VEGF by laser-scanning confocal microscopy in double staining.

We found that leptin immunoreactivity was present in areas that were also Lyve-1 and VEGF positive but leptin staining was generally much more marked than Lyve-1 and VEGF labeling (data not shown). VEGF immunoreactivity was also observed inside Ob-R positive areas.

## Discussion

In the current study, we identified leptin and Ob-R expression in epithelial cells and in structures within the *lamina propria* of the large intestine mucosa from IBD and control patients. Then, we also identified GLUT5 immunoreactivity in specific *lamina propria* areas where leptin and /or Ob-R were expressed.

### Distribution of leptin and Ob-R in the epithelium of the large intestine

Previous works have shown the expression of leptin and Ob-R in epithelial cells of normal and cancer colon using various methods (Western blotting, RT-PCR and immunohistochemistry). In porcine tissue, leptin was reported to be present in the colon with a much higher level of expression along the basal side of the colonocytes when compared to the lateral side and with an amorphous appearance at the apical surface (Hansen et al. 2008). In the human colon, leptin and Ob-R were detected on the cell membrane and in the cytoplasm of colonocytes, probably located in the Golgi apparatus (Hardwick et al. 2001). Leptin was also found to be present in colonic samples of IBD patients but absent in those of healthy subjects. Conversely, Ponemone et al. (2010) reported leptin expression in colonic epithelial cells in both control and IBD patients.

In the current study, we reported that epithelial colonic cells showed apical staining for both leptin and Ob-R. However, we found that there is a substantial difference between them: leptin staining was limited to short apical epithelial portions of some samples, whereas Ob-R was detected on the brush border membrane of the entire epithelium of all samples. This detection is in agreement with the immunohistochemical localization of the long leptin receptor found in the brush border plasma membrane of human duodenum in which immunostaining occurred in all enterocytes (Barrenetxe et al. 2002).

Occasionally, we also observed leptin and Ob-R immunoexpression in the cytoplasm and/or basolateral membrane of epithelial cells. The intra-cytoplasmic staining of leptin and Ob-R could reflect their internalization in endosomes after ligand-binding or a cytoplasmic pool storage, whereas the basolateral localization could be indicative of Ob-R/ligand complexes that are formed from leptin coming from vessels or cells in the connective tissue.

On the basis of these different distributions, we speculate that the expressions of Ob-R and leptin that were present occasionally and with variable distribution among the samples represent a transient expression, locally induced or modulated by stimuli or physiological conditions of the sample. A similar observation was reported for Ob-R expression in Caco-2/15 cells (Cammisotto et al. 2012). On the contrary, we believe that our Ob-R detection on the brush border membrane of the epithelium, which was a constant finding in all samples, may be a constitutive location representing a strategic position for monitoring leptin in the intestinal luminal content. These assumptions can account for the unequal literature data on the epithelial expression of leptin and Ob-R.

The localization of Ob-R both on the apical and basolateral membrane of colonocytes raises the interesting question of whether functionally distinct apical and basolateral membrane domains are present in colonocytes. Evidence of a functional complex leptin/Ob-R has been described only at the basolateral and not at the luminal surface of porcine enterocytes and colonocytes, although apical labeling for Ob-R was detected in both cell types (Hansen et al. 2008). In support of a luminal action, it has been shown that the luminal effect of leptin in the colon is different from that produced by systemic leptin. For example, in rats, systemic leptin stimulated proliferation of epithelial cells in the right colon but not in the left, whereas intracolonic infusion of leptin did not exert a proliferative effect on colonic epithelial cells and did not modify leptinemia (Aparicio et al. 2004). Likewise, experiments with polarized cell lines have provided clear evidence that the effect of leptin critically depends on the apical or basolateral origin of the stimulus (Cammisotto et al. 2012).

Our detection of Ob-R on the apical membrane of colonic cells indicates a possible action of the luminal leptin. Although our antibody recognizes all Ob-R isoforms, the functional Ob-Rb isoform is present on the apical membrane of colonic cells as suggested by others (Barrenetxe et al. 2002; Aparicio et al. 2004). In addition, it has been demonstrated that inflamed colonic epithelial cells are able to produce and release leptin into the intestinal lumen and that rectal application of leptin induces epithelial wall damage and intestinal inflammation (Sitaraman et al. 2004). The authors have suggested that luminal leptin may serve in inflamed colonic samples from UC patients to diffuse inflammation by acting, like other proinflammatory cytokines, to activate nuclear transcription factors NF-κB and AP-1, which are implicated in the pathogenesis of IBD.

Whether leptin is produced by epithelial cells or is taken up from the luminal colonic content is still a controversial matter and studies in this regard have not yielded conclusive results. In rats, a leptin secretory function for colonocytes has not yet been confirmed although leptin has been detected in luminal colonic content at a concentration level capable of activating Ob-R (0.3 nmol/l) and with a higher level in normally fed rats than in fasted rats (Plaisancie et al. 2006).

In humans, the presence of leptin in the colon’s lumen has been described only in a few studies that have produced different explanations about its origin. There is evidence that an amount of stomach-secreted leptin reaches the colon without being degraded (Sobhani et al. 2002), which suggests that the stomach is a reliable source of leptin in the digestive tract (Bado et al. 1998; Sobhani et al. 2000; Buyse et al. 2002). Otherwise, an alternative source is represented by colonic cells. Sitaraman et al. (2004) demonstrated that the leptin concentration was 15-fold greater in the colonic lavage from patients with UC or CD, when compared to non-IBD patients, identifying the inflamed colonic epithelial cells as a possible source of leptin in the luminal content of IBD patients. By immunohistochemistry, the same study confirmed a strong leptin staining at the subapical surface of the colonic epithelial cells from UC patients and detected its absence in epithelial cells from normal colon, despite the fact that functional leptin concentration of leptin was found to be released in colonic lavages from normal colonic cells (Sitaraman et al. 2004).

Here, we observed that the epithelial expression of leptin and Ob-R showed no substantial differences between the samples obtained from the proximal and distal tract of the large intestine and between the IBD and CTRL patient groups. Because of their limited expression on the epithelium, we were unable to compare their expression across the three patients’ groups.

### Distribution of leptin and Ob-R in the *lamina propria* of the large intestine

An important finding of our study is the immunolocalization of leptin and its receptor in subepithelial structures of the *lamina propria* in human colonic samples, showing their further expression with respect to well-known localization in the epithelium. The presence of a leptin system in the *lamina propria* indicates not only a possible peripheral function but also strongly argues for endocrine/paracrine leptin action, as the vessels and main cells involved in the inflammatory response, such as mast cells, lymphocytes and macrophages, are present in the *lamina propria*.

Our report is the first to describe the simultaneous localization of leptin and Ob-R in immunolabelled areas of *lamina propria* in the samples from IBD and control patients. This staining was detected without important differences between the inflamed and non-inflamed *status* of the tissues. However, as previously observed with epithelial staining, both patterns of expression were variable for intensity and distribution among the samples. By comparing the localization of leptin with that of Ob-R in double-immunofluorescence staining and corresponding areas of adjacent sections, we observed that their expression was colocalized in cells and vessels, although extensive immunoreactive areas for leptin often did not coincide with those labeled for Ob-R, indicating that leptin and its receptor are not necessarily located in the same zone. Thus, it is possible that different reactive microenvironments may be a key factor in the regulation of their expression and observed differences in the labeling intensity. It could also be that the leptin systems of the epithelium and *lamina propria* may interact with each other; consequently, their effects depend on the balance of the two compartments.

In mammals, previous leptin detection in *lamina propria* has been reported in porcine transverse colon (Hansen et al.^2008^) and in intestinal lymphocytes of mice (Siegmund et al. 2004), whereas Ob-R was detected in *lamina propria* immune cells of the rat’s small intestine (Lostao et al. 1998) and in perivascular submucosal cells of murine small and large intestine (Rajala et al. 2014).

In humans, only a previous study reported immunohistochemical presence of leptin in the *lamina propria* from colonic samples of both control, UC and CD patients, showing a more intense leptin reactivity in active IBD, compared to inactive (Ponemone et al. 2010).

Recent studies have further shown the immunohistochemical expression of leptin and Ob-R in the *lamina propria* from colonic mucosa of patients with diarrhea-predominant irritable bowel syndrome (IBS-D; Liu et al. 2018) and colorectal cancer (Al-Shibli et al. 2019). Consistent with these data, our results further support the theory that localization of leptin and its receptor in the mucosa of the large intestine may be a histological finding that characterized samples in particular pathological/physiological conditions. Moreover, this localization can have important implications because it is indicative of a paracrine role of leptin in the *lamina propria*. Indeed, modulatory effects of leptin on immune cells of the gastrointestinal tract have been widely investigated in mice, showing that leptin stimulates the release of IL-18 by mononuclear cells of the *lamina propria* in experimental colitis of leptin-deficient *ob/ob* mice (Siegmund et al. 2002) and exerts, through the modulation of Ob-R expression on ileum epithelial cells, as well as in cells of the *lamina propria*, an inflammatory effect in *Clostridium Difficile* toxin A-induced enteritis (Mykoniatis et al. 2003). In addition, reduced levels of cytokines and chemokines, as well as neutrophil infiltration were observed in colonic tissue of leptin-deficient *ob/ob* mice, in comparison with WT mice and a selective deficiency of the functional leptin receptor on the T lymphocytes was responsible for the delayed development of intestinal inflammation, suggesting a local action of leptin in regulating intestinal inflammation (Siegmund et al. 2002).

In humans, Ob-Rb expression was detected on colonic dendritic cells from normal and CD tissues, mainly localized below the epithelium and more abundant in the inflamed than non-inflamed tissues (Al-Hassi et al. 2012). Recently, Liu et al. (2018) reported that the activation of leptin and Ob-R-expressing mast cells promote an immune process in the intestinal mucosa and the mast cell activation rate is significantly increased in IBS-D patients, when compared to controls. In a double-labeling immunofluorescence study, they showed the colocalization of leptin and Ob-R on mast cells scattered in the *lamina propria* and submucosa of colonic biopsies from IBS-D patients.

On the basis of these findings, the most probable hypothesis for the interpretation of our data in the *lamina propria* is that the expression of leptin may be related to inflammation processes present in the samples. However, we observed leptin and Ob-R expression in inflamed and non-inflamed colorectal biopsies from IBD and CTRL patients. In both the proximal and distal tracts, the number of leptin-positive samples was higher than Ob-R-positive samples in all groups, except non-inflamed samples from the distal tract in UC.

A similar percentage of samples expressing leptin or Ob-R was also observed across all three patients’ groups when classified by BMI.

By comparing samples for both expressions, the inflammatory mucosal *status* of the sample did not appear to be conditioned, as we observed Lept^+^/Ob-R^+^ samples both in inflamed and non-inflamed tissues and in all patient groups. The percentage of (Lept^+^/Ob-R^+^) samples was higher than those with a single positive expression (Lept^+^/Ob-R^−^ or Lept^−^/Ob-R^+^) or with the absence of both expressions (Lept^−^/Ob-R^−^), suggesting leptin as a founder event for Ob-R expression. Indeed, in the UC group, we observed that all inflamed Ob-R^+^ samples and non-inflamed Ob-R^+^ samples of the proximal tract were significantly correlated to Lept^+^ samples, implying that a local modulation of Ob-R expression may be closely related to leptin-positive zones. A similar correlation is also true for inflamed distal samples from CD patients.

We showed here that the leptin- and Ob-R-positive areas in the *lamina propria* were also immunoreactive for GLUT5. In the UC group, inflamed leptin^+^ or Ob-R^+^ samples of the distal tract were significantly correlated to GLUT5^+^ samples. Likewise, the percentage of Lept^+^/Ob-R^+^/GLUT5^+^ samples was higher when compared to other possible combinations especially in tissues from the distal part of the large intestine in the UC and CTRL patient groups. GLUT5 is a high-affinity fructose transporter whose expression is mainly induced by the presence of fructose but the regulatory pathways that modulate its expression are largely unknown (Charrez et al. 2015).

In rodents, it has been demonstrated that systemic or luminal administration of leptin increases mucosal absorptive capacity for carbohydrates. In particular, in a rat model of short bowel syndrome, systemic infusion of leptin was found to enhance small intestine carbohydrate absorption and GLUT5 gene expression beyond the normal adaptive response (Pearson et al. 2001). Similarly, infusion of luminal leptin was positively associated with an increased activity of GLUT5 transport, whereas oral fructose has been found to be associated with leptin release in the intestinal lumen without inducing a change in the plasma leptin levels (Sakar et al. 2009).

In a previous study, we found GLUT5 expression in clusters of lymphatic vessels in the colonic mucosa from the same IBD and control patients used in this study. We considered the GLUT5-immunoreactive areas “as an impaired pattern of lymphatic capillary networks, probably related to proliferative zones” (Merigo et al. 2018). As highlighted by many reports, angiogenesis and lymphangiogenesis are hallmark features of chronic gut inflammation (Koutroubakis et al. 2016). Consequently, the extensive expression of the leptin system in the same areas of the *lamina propria*, in which we detected the presence of aberrant lymphatic vessels, could indicate a link between fructose and leptin for the growth and proliferation of vessels. It could be that the action of the leptin system is primary with respect to the action of GLUT5 in these areas, given the greater percentage of leptin-positive samples compared to GLUT5 both in the UC and CRTL group. But, it could be mostly a concomitance of effects: the changes induced in the local microenvironment could in turn exert beneficial or adverse effects depending on the context in which they occur. A beneficial effect of an agent can become adverse in a pathophysiologic contest such as obesity, inflammation, or neoplastic conditions. Therefore, we believe that a local disequilibrium of different processes could justify the heterogeneous expressions of leptin and Ob-R observed in our samples.

Furthermore, we found that the combined expressions of leptin, Ob-R and GLUT5 in CD were different in comparison to the other groups. In particular, in non-inflamed samples from the distal tract, the percentage of Lept^+^/Ob-R^+^ samples that lacked GLUT5 expression was greater than the GLUT5 positive samples. This conflicting finding may be due to a limited numbers of samples in CD that may not be representative of the whole group or may be the result of the distinct immunological, pathological and clinical profiles that characterize CD, as opposed to UC.

With regard to the presence of immunoreactive areas in the control samples, it could be said that part of our control group does not represent a completely healthy population, consisting of subjects without IBD diagnosis but with other clinical problems, such as chronic diarrhea or abdominal pain. Thus, IBS cannot be excluded for these patients. There is evidence of increased cellularity, particularly mast cells, in IBS patients’ *lamina propria*, when compared to non-IBS control (Piche et al. 2008) and the fact that the immunoreactive areas, even in non-inflamed samples may represent an undetected, low-grade subclinical inflammation, also present in patients that do not reveal other colonic mucosal abnormalities should not be underestimated. An increase in *lamina propria* immune cells, even in colonic mucosa of patients with normal colonoscopy and conventional histologic interpretation has been previously described (Salzmann et al. 1989).

Here, we also found a partial overlap between leptin/Ob-R expression and markers of lymphatic (LYVE-1) and endothelial (VEGF) vessels, suggesting that leptin and its receptor may have a role in vascular function. It could be that the immunoreactive areas identified within the *lamina propria* in this study represent new structures with architectural modifications, probably due to a reorganization of the lymphatic or vascular system with the participation of immune cells. Ectopic lymphoid aggregates with such characteristics have already been previously indicated as centers capable of initiating a local immune response (Becker et al.^2014^). In support of our hypothesis, Rajala et al. (2014) recently identified, by immunohistochemical analysis of the small and large bowels in mice, “an unknown population of Ob-Rb^+^ cells” that were in close association with the vascular structures within the intestinal submucosa. No overlap between the submucosal Ob-Rb cells and endothelial and lymphatic markers was observed, suggesting that they represent a population of atypical pericytes with a potential role in the regulation of leptin-induced mechanisms.

Finally, we cannot exclude that the expression of leptin observed in our samples may play a role in the interactions between the host and the microbiota. Indeed, it has been demonstrated that leptin regulates both antimicrobial peptides and microbiota composition, acting with a mechanism, not well-defined, although independent of both food parameters and of its direct effect on gut epithelium (Rajala et al. 2014).

A variety of studies have focused their attention on the role of gut microbiota on the pathogenesis of IBD and an association between dysbiosis and IBD is widely documented, even though a cause and effect relationship between them is yet to be proven and the overarching question of whether dysbiosis precedes the development of IBD, or is a consequence of the inflammation, has not yet been answered (Haag and Siegmund 2014; Ni et al. 2017).

In conclusion, we demonstrated that leptin and Ob-R are expressed heterogeneously in colonocytes and *lamina propria* structures of colorectal samples, suggesting different sites of action of leptin and Ob-R in the large intestine mucosa. Additionally, we showed that leptin and Ob-R expression in the *lamina propria* does not reflect the *status* of mucosa inflammation being present in both inflamed and non-inflamed tissues from IBD and control patients, indicating that mucosal abnormalities related to the leptin system may remain undetected at a subclinical level.

Moreover, we found that the immunoreactive areas for the leptin system in the *lamina propria* are also positive for GLUT5, a fructose transporter overexpressed in clusters of lymphatic vessels. We retain that the immunoreactive areas represent new structures and, similar to that which happens in the cancer pathway, a “cross-talk” between epithelial cells and the connective microenvironment, can also occur in colonic mucosa, with consequent functional integration of multiple signaling pathways (Okayasu et al. 2009; Okayasu 2012).

Our results provide a characterization of the expression of leptin and Ob-R that represents the necessary basis for future studies. Due to absence of validated biomarkers of IBD disease monitoring, our finding provides a useful tool to monitor the status of the colonic mucosa using identification of Lept, Ob-R and GLUT5 molecules.
